# (PVA/Chitosan/Fucoidan)-Ampicillin: A Bioartificial Polymeric Material with Combined Properties in Cell Regeneration and Potential Antibacterial Features

**DOI:** 10.3390/polym11081325

**Published:** 2019-08-09

**Authors:** Andres Bernal-Ballen, Jorge-Andres Lopez-Garcia, Kadir Ozaltin

**Affiliations:** 1 Grupo de Investigación en Ingeniería Biomédica, Vicerrectoría de Investigaciones, Universidad Manuela Beltrán, Avenida Circunvalar No. 60-00, Bogotá 110231, Colombia; 2 Centre of Polymer Systems, Tomas Bata University in Zlín, Tr. Tomase Bati 5678, 76001 Zlín, Czech Republic

**Keywords:** bioartificial polymeric material, cell proliferation, chitosan, fucoidan, polyvinyl alcohol, ampicillin

## Abstract

Chitosan, fucoidan, and polyvinyl alcohol are categorized as polymers with biomedical applications. Ampicillin, on the other hand, is considered as an important antibiotic that has shown effectivity in both gram-positive and gram-negative micro-organisms. The aforementioned polymers possess unique properties that are considered desirable for cell regeneration although they exhibit drawbacks that can affect their final application. Therefore, films of these biomaterials were prepared and they were characterized using FTIR, SEM, XRD, degree of swelling and solubility, and MTT assay. The statistical significance of the experiments was determined using a two-way analysis of variance (ANOVA) with *p* < 0.05. The characterization techniques demonstrated that the obtained material exhibits properties suitable for cell regeneration, and that a higher concentration of natural polymers promotes cells proliferation to a greater extent. The presence of PVA, on the other hand, is responsible for matrix stability and dictates the degree of swelling and solubility. The SEM images demonstrated that neither aggregations nor clusters were formed, which is favorable for the biological properties without detrimental to the morphological and physical features. Cell viability was comparatively similar in samples with and without antibiotic, and the physical and biological properties were not negatively affected. Indeed, the inherent bactericidal effect of chitosan was reinforced by the presence of ampicillin. The new material is an outstanding candidate for cell regeneration as a consequence of the synergic effect that each component provides to the blend.

## 1. Introduction

Biomedical engineering applies and develops therapies and technologies in order to support, repair or replace damaged cells, tissues, and organs, and it combines techniques from diverse disciplines such as physics, chemistry, biology, engineering, and medicine [1]. Thus, in the past decades a new generation of synthetic biodegradable polymers and analogous natural polymers have been developed for biomedical applications [2]. However, natural polymers do not have the appropriate mechanical properties whereas synthetics are deficient in terms of biocompatibility [3]. Thus, in order to overcome the poor performance of natural polymers, bioartificial polymeric materials (BAPMs) have been introduced [4,5,6,7,8]. These combine the biocompatibility of the biological component with the physical and mechanical properties of the synthetic ones [9]. BAPMs may be produced as hydrogels, films, scaffolds, and a great variety of potential applications have been reported including dialysis membranes, artificial skin, cardiovascular devices, implants, bandages, or even controlled drug-release systems [10,11]. 

Natural polymers possess several inherent advantages, such as bioactivity, the ability to present receptor-binding ligands to cells, susceptibility to cell-triggered proteolytic degradation, and natural remodeling. Synthetic biomaterials, on the other hand, are generally biologically inert, they have more predictable properties and batch-to-batch uniformity, and they have the unique advantage of having tailored property profiles for specific applications, hence, they are devoid of many of the disadvantages of natural polymers [2]. 

One of the main biopolymer used in biomedical applications is chitosan (CHI) [12,13,14]. This polysaccharide is primarily composed of β-(1–4)-linked d-glucosamine and *N*-acetyl glucosamine subunits. It is biodegradable, biocompatible, non-toxic, and it has a functional hydrophilic surface that promotes cell growth. Moreover, it exhibits antimicrobial activity, a characteristic that is useful for tissue engineering applications [15]. Reports that describe CHI used in cell adhesion, proliferation, and differentiation are plentiful in the literature [16,17]. Another important feature of CHI consists of its significant osteoconductivity, and minimal osteoinductivity. This induces the proliferation of osteoblasts, and neovascularization in vivo. For orthopedic applications, CHI can be prepared in various geometries such as sponges, fibers, films and other complex structures. In addition, this material has shown antibacterial, antifungal, hemostatic, analgesic, and mucoadhesive properties, all of which are relevant in drug delivery and tissue healing [18]. CHI satisfies most of the properties that a material should have as a candidate for tissue engineering applications [19]. 

Another relevant material in the field of biomedical application is fucoidan (FUC). This natural polymer is a marine-sourced sulphated polysaccharide extracted from brown algae, and it has numerous biological activities including anticancer, antitumor, antivirus, and anti-inflammatory properties [20,21]. Indeed, it is highly water soluble, biocompatible, biodegradable, and non-toxic [22] and it has demonstrated its antiviral activities both in vivo and in vitro. Since it presents low cytotoxicity compared with other antiviral drugs, FUC is currently used in clinical medicine [23]. FUC has been targeted in tissue engineering applications; also, it triggers the biological activities of alkaline phosphatase and osteocalcin, which are phenotypic markers for the early stages of osteoblast differentiation [16]. 

In spite of the merits that polysaccharides exhibit as biomaterials, they suffer from several drawbacks including variations in material properties based on their source, microbial contamination, uncontrolled water uptake, poor mechanical strength, and unpredictable degradation patterns [24]. Nonetheless, these polymers are readily recognized and accepted by the body due to their biochemical similarity with components of the human extracellular matrix [18]. 

Synthetic polymers are an appropriate counterpart for reducing the drawbacks of natural polymers. For that reason, these materials have become an obvious alternative for biomedical applications. There are three explanations for this: although most biologically derived biodegradable polymers possess good biocompatibility, some may trigger an immune response in the human body that could possibly be avoided by the use of an appropriate synthetic biopolymer; chemical modifications to biologically derived biodegradable polymers are difficult; and chemical modifications likely cause alteration in the bulk properties of biologically derived biodegradable polymers [25]. 

Polyvinyl alcohol (PVA), a representative water-soluble polymer, is considered as one of the most attractive biomedical polymers due to its synergistic properties such as biocompatibility, excellent mechanical strength, flexibility, thermal stability, and absence of toxicity [26]. A complete description of this material can be found in numerous scientific publications [26,27,28,29,30,31,32]. Indeed, blending PVA with a great variety of natural polymer is also reported [33,34,35,36,37,38]. These publications describe the physical, chemical, and biological interactions of PVA, and most of the cited publications mention the use of PVA in blends that have potential uses in the biomedical field. 

The combination of natural and synthetic polymers has emerged as a plausible solution for overcoming the inherent weaknesses exhibited by natural materials [39]. Ideally, the blend is not detrimental to the mechanical properties of the synthetic polymer, as evidenced in a profusion of published literature [12,39,40,41,42,43]. In fact, it has been shown that the variation in the concentration of the blend is a crucial factor for obtaining a material with appropriate mechanical properties [12], without negatively affecting the biological properties [8]. Furthermore, if BAPMs had antibacterial properties this would be a desirable feature with positive repercussions in the bioengineering field. In this matter, a previous study has shown that a low concentration of ampicillin (AMP) in a matrix made of CHI and PVA showed moderate antibacterial activity against bacterial strains, even without adding an extremely high concentration of the antibiotic [3]. 

The combination of PVA and CHI has been fully described in the literature. There are also reports in which these polymers are combined with antibiotics [44]. Nonetheless, the system of PVA, CHI, FUC, and AMP has not been described to date, and we hypothesize that this BAPM is a new approach to cell regeneration because the BAPM combines the recognized biological properties of CHI and FUC, the outstanding mechanical features of PVA, and the antibacterial activity of AMP. Therefore, this work involves the preparation of a new BAPM consisting of PVA/CHI/FUC/AMP, the characterization of the obtained material, and an in vitro evaluation of its performance for cell culture. 

## 2. Materials and Methods 

### 2.1. General Information

Poly(vinyl alcohol) (PVA) (M_w_ = 47,000 g mol^−1^), with a polymerization degree of 1000 and 98% of hydrolysis was provided by Fluka Analytica (Prague, The Czech Republic); chitosan (CHI) of medium molecular weight with a deacetylation degree of 75–85%, and glutaraldehyde (GLU) grade II at 25% in H_2_O were purchased from Sigma Aldrich (Prague, The Czech Republic). Anticoagulant fucoidan (FUC) was obtained from *Fucus vesiculosus* and provided by Sigma Aldrich, St. Louis, MO, USA. Lactic acid (analytical grade) was produced by Lachema, Brno, The Czech Republic; and hydrochloric acid (analytical grade) was supplied by Penta, Prague, The Czech Republic. Sodium ampicillin (AMP) was produced by Farmalógica, S.A. (Bogota-Colombia) and donated to this research by the Hospital Cardiovascular del Niño de Cundinamarca (Soacha, Colombia). None of the reagents were subject to further processing.

### 2.2. Sample Preparation

A 1% w/v acid solution of CHI was prepared by the addition of the polymer to acetic acid at 0.5 M under mild manual stirring. Once the polymer was partially dissolved, the solution was stirred in a shaker (Multifunctional Orbital Biosan, PSU 20i, Latvia) for 24 h. An aqueous solution of PVA at 1% w/v was prepared in deionized water at 70 °C under vigorous magnetic stirring using a Heidolph MR Hei-Standard magnetic stirrer with heating (Heidolph Instruments GmbH, Schwabach, Germany). Natural and synthetic polymers solutions were crosslinked and plasticized using glutaraldehyde-hydrochloric acid (0.25 wt% and 1.2 wt%) and lactic acid (0.5 wt.%) as compared to the total amount of the polymer. The prepared solutions underwent degasification using an ultrasonic cleaning unit (S80 Elmasonic, Elma GmbH, with degasification function, Singen, Germany). An aqueous solution of fucoidan (FUC) was prepared by dissolving the polymer in distilled water to obtaining a 0.1% w/w solution. Ampicillin was dissolved in distilled water using a mild magnetic stirrer (Heidolph Instruments GmbH, Schwabach, Germany). 

CHI and FUC were blended in a ratio of 1:1 v/v and the new solution was stirred for 30 min at 600 rpm. Once the solutions of natural polymers were obtained, the artificial polymer was added and stirred for 30 more minutes at 600 rpm followed by the addition of AMP at 1.0 wt% of the total amount of the polymers. The solutions were stirred for 10 min and films were obtained using the casting method and pouring 0.5 mL per cm^2^ on plastic Petri dishes. Films were allowed to dry at 37 °C for a week in a no-air circulating oven. Table 1 shows the prepared films.

### 2.3. Fourier Transform Infrared Spectroscopy (ATR-FTIR) 

Spectra for pure polymers and all blends were obtained using a Nicolet iS5 spectrometer (Thermo Fisher, Waltham, MA, USA) equipped with an attenuated total reflectance (ATR) accessory utilizing the Zn-Se crystal. Each spectrum represents 64 co-added scans referenced against an empty ATR cell spectrum. The spectra range was from 4000 to 650 cm^−1^ with a resolution of 1.92 cm^−1^.

### 2.4. X-ray Photoelectron Spectroscopy (XPS) 

Determination of the chemical composition of the blends was carried out by X-ray photoelectron spectroscopy (XPS). The Thermo Scientific K-Alpha XPS system (Thermo Scientific, UK) was used, which was equipped with monochromatic Al K-alpha X-ray source (1486.6 eV) with an X-ray beam of 400 mm in size at 6 mA 12 kV. The spectra were collected in the constant analyser energy mode with pass energy of 200 eV and Thermo Scientific Avantage 5.952 software (Thermo Fischer Scientific) was used for digital acquisition. 

### 2.5. Scanning Electron Microscopy (SEM) 

Micrographs of the prepared samples were taken by the scanning electron microscope Nova NanoSEM 450 (FEI, Hillsboro, OR, USA) equipped with a high vacuum detector and operated at 5 kV with a spot size of 2.5 A coating with a thin layer of gold/palladium was performed by a sputter coater SC 7620 (Quorum Technologies, Newhaven, East Sussex, UK). 

### 2.6. Degree of Swelling and Solubility

The gravimetric method was used to obtain the degree of swelling and solubility. Disk specimens were dried in a desiccator for 24 h to obtain their dry weight (*W*_1_). Then, the samples were immersed in deionized water at 20 °C for several different time periods (2, 4, 6, 8, 10, 15, 20, 30, 60, 90 min and 24 h). At the end of each period, the excess water was removed from the surface with filter paper and the specimens were weighed again. The last measure (24 h) was considered as the swelling limit (*W*_2_) because in that period the equilibrium was reached. Finally, samples were dried until they achieved constant weight (*W*_3_). The degree of swelling (DS) and degree of solubility (SD) were determined using Equations (1) and (2), respectively. The test was performed with five specimens for each sample (n = 5) to obtain statistically significant data:

DS = [(*W*_2_ − *W*_1_)/*W*_1_] × 100 (1)

SD = [(*W*_1_ − *W*_3_)/*W*_1_] × 100 (2)

### 2.7. Cell Proliferation Test (MTT) 

Prior to the in vitro cytocompatibility test, samples were placed in 10 × 10 mm foil and subjected to UV-radiation source (wavelength of 258 nm) for 30 min. Primary mouse embryonic fibroblast cells (NIH/3T3, ATCC^®^ CRL-1658^TM^, USA) were used as a cell line. The ATCC-formulated Dulbecco’s Modified Eagle’s Medium (BioSera, France) containing 10% of calf serum (BioSera, France) and 100 U mL^−1^ Penicillin/Streptomycin (BioSera, France) was used as a culture medium. The cells were seeded onto the samples in a concentration of 2 × 10^4^ cells per mm^2^ and placed in an incubator for 72 h at 37 °C. All the tests were performed in triplicate.

After the incubation period of 72 h, the cell viability was tested using the MTT assay (Duchefa Biochemie, Netherlands). Firstly, the cells were washed with PBS (BioSera, France) and fresh medium with MTT with a concentration of 0.5 mg/mL was added. After 4 h, formed formazan crystals were dissolved in DMSO and the absorbance was measured at 570 nm and the reference wavelength was adjusted to 690 nm. The results are presented as reduction/augmentation of cell viability as a percentage when compared to cell cultivated on pure PLA.

### 2.8. Statistical Analysis

All experiments with quantitative measurements were performed on five specimens, except for the cell culture where three specimens were tested. The experimental values are reported in the form of average ± standard deviation. Results were statistically compared by applying a two-way analysis of variance (ANOVA) with *p* < 0.05 with SPSS software.

## 3. Results and Discussion

### 3.1. Attenuated Total Reflectance Fourier Transform Infrared Spectroscopy (ATR-FTIR)

**Figure 1:**
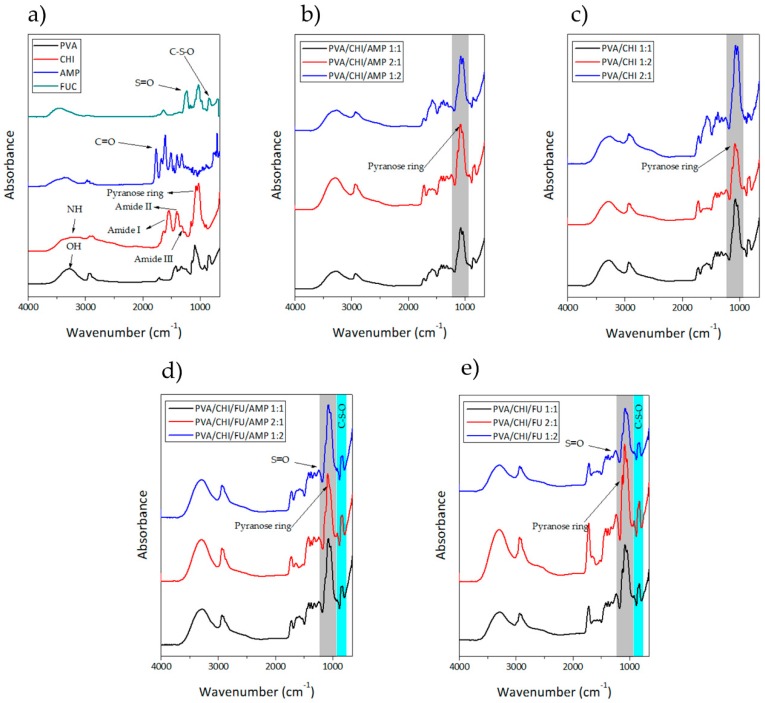
FTIR for the obtained BAPM. (**a**) Row materials; (**b**) PVA/CHI/AMP samples; (**c**) PVA/CHI samples; (**d**) PVA/CHI/FUC/AMP samples; and (**e**) PVA/CHI/FUC samples.

Figure 1 shows the obtained spectra for the BAPMs as well as for the individual components. For PVA, an intense band between 3550 and 3200 cm^−1^ appeared and it corresponds to OH from the intermolecular and intramolecular hydrogen bonds. The detected transitions around 2900 cm^−1^ are associated to C–H from alkyl groups [45]. At 1423 cm^−1^ the –CH_2_– bending appears, and at 1416 and 1327 cm^−1^ the band ascribed to CH and OH, respectively, is evident [45,46,47]. The peak at 848 cm^−1^ corresponds to skeletal C–H rocking of pure PVA [48].

The spectrum for FUC has been elucidated in the literature and these reports are in a good agreement with our results. The peaks at 3470 and 2936 cm^−1^ correspond to the stretching vibration of hydrogen bond for OH and C–H groups, respectively. The bands located between 1200 cm^−1^ and 970 cm^−1^ are caused by C–C and C–O stretching vibrations in the pyranoid ring and C–O–C stretching of the glycosidic bonds, whereas the absorption band located at 1240 cm^−1^ belongs to S=O stretching and evidences the presence of sulfate. An absorption peak at 1648 cm^−1^ is attributed to the C=O asymmetric stretching vibrations of the carbonyl groups and the peak centered in the region of 840 cm^−1^ and the shoulder at 820 cm^−1^ (C–S–O) suggest a complex pattern of substitution, primarily at the C–4 position with other substitutions at C–2 and/ or C–3 (equatorial positions) in lower amounts. The features at 622 cm^−1^ and 583 cm^−1^ can be ascribed to the asymmetric and symmetric O=S=O deformation of sulfates [49,50,51,52,53].

AMP shows an absorption peak in the region of 1730–1720 cm^−1^, which is caused by C=O β-lactam stretching. The peaks at 1664 and 1560 cm^−1^ belong to C=O amide stretching and –NH amide groups, respectively [54]. 

PVA and CHI interactions are evidenced in the frequency observed in the region of 3388 cm^−1^ to 3427 cm^−1^ corresponding to intermolecular hydrogen bonds [36]. Blends of PVA/CHI show an absorption band located between 1640 and 1560 cm^−1^, which is connected to symmetric stretching and bending of acetamide groups, respectively. The change in the characteristic shape of the CHI spectra, as well as the shifting of peak to a lower frequency range on account of hydrogen bonding between –OH of PVA and –OH or –NH_2_ of chitosan were observed in the blended films. These results suggested the formation of hydrogen bonds between the CHI and PVA molecules [3].

The combination of CHI and FUC is mediated by an electrostatic interaction of the positively charged amino group in CHI and the negatively charged sulfate group in FUC [55]. For that reason, the characteristic peak of sulfate in FUC located at 1240 cm^−1^ is not evidenced in the samples containing FUC. The peak at 1725 cm^−1^ confirmed the formation of CHI/FUC complex. Moreover, the peak corresponding to the cross-linking primary amine of CHI is centered at 1530 cm^−1^ [56]

Peaks for CHI are distinguishable at particular wavenumbers. Thus, the NH group-stretching vibration appears at 3375 cm^−1^, and the signal for OH is located at 3450 cm^−1^ [57]. The symmetric, and asymmetric –CH_2_– stretching occurring in the pyranose ring are located at 2920, 2880, 1430, 1320, 1275 and 1245 cm^−1^ [58]. The amide I, II, and II vibrations are located at 1650, 1586 cm^−1^, and 1322 cm^−1^, respectively. The saccharine-related signals are at 1155 and 900 cm^−1^ [59,60]. The peaks at 1030 and 1080 cm^−1^ indicate the C–O stretching vibration [5].

### 3.2. X-ray Photoelectron Spectroscopy (XPS) 

The surface chemical compositions of the individual components are given in Table 2. The main components of PVA, CHI, FUC and AMP are carbon and oxygen, therefore the C1s and O1s have the highest atomic levels. Furthermore, samples with a higher ratio of CHI present a higher amount of carbon and a relatively lower amount of oxygen due to their molecular formula, except for the PVA/CHI/FUC 1:2 counterpart. The level of nitrogen shown for each sample is due to the presence of CHI and AMP. The samples that have a higher ratio of CHI show the highest N1s level, compared to other samples, except for the PVA/CHI/AMP 2:1 counterpart only. The level of sulfur stems from AMP and FUC due to their molecular formula. Thus, the S2p level observed for the samples containing AMP and FUC was expected. However, the level is low since both are additives and not the main components of the prepared films. The reason for the unexpected behavior of the PVA/CHI/FUC 1:2 sample with regard to the C1s level and the PVA/CHI/AMP 2:1 sample for N1s is unclear and needs to be studied further [61,62,63,64,65]. 

**Figure 2:**
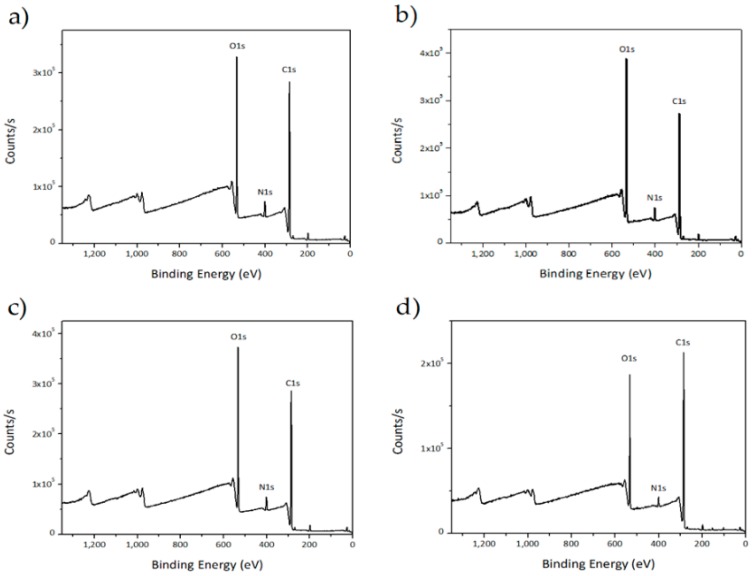
XPS spectra for (**a**) PVA/CHI/AMP 1:2; (**b**) PVA/CHI/ 1:2; (**c**) PVA/CHI/FUC/AMP 1:2; (**d**) PVA/CHI/FUC 1:2.

Figure 2 shows the representative XPS spectra analysis for the components with and without AMP containing counterparts with a 1:2 ratio. As can be seen, all representative samples are similar for C1s, N1s and O1s spectra because the selected components have the same PVA/CHI ratio. The C1s spectra show three bonding states at 283.08 eV attributed to C–C and C–H bonds; at 285.08 eV attributed to C–NH and C–NH_2_ bonds; and at 286.08 attributed to C–O–C=O and C–OH bonds, due to the presence of PVA and CHI. The O1s spectra exhibit a major bonding state at 536.08 eV attributed to C–O bond, due to the presence of PVA and CHI. The rather lower level of N1s spectra was detected at 399 eV attributed to CHI and AMP. The S2p spectra were not sufficient to appear in Figure 2, which was related to AMP and FUC, however, their amounts are presented in Table 2. 

### 3.4. Scanning Electron Microscopy (SEM) 

**Figure 3:**
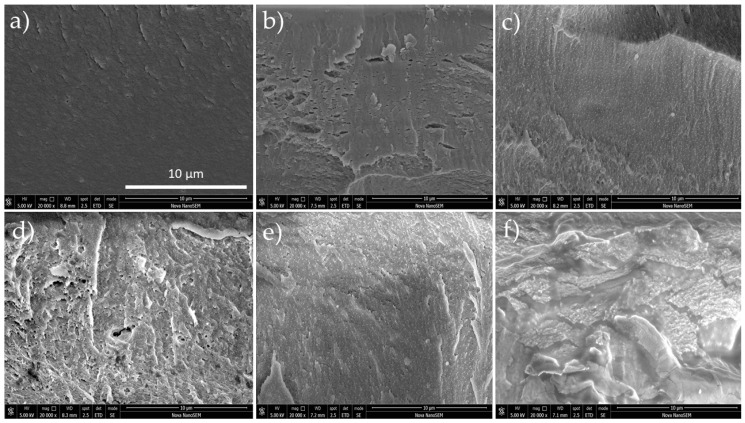
SEM images for BAPM in the presence of AMP. (**a**) PVA/CHI/AMP 1:1; (**b**) PVA/CHI/AMP 2:1; (**c**) PVA/CHI/AMP 1:2; (**d**) PVA/CHI/FUC/AMP 1:1; (**e**) PVA/CHI/FUC/AMP 2:1; and (**f**) PVA/CHI/FUC/AMP 1:2.

The surface morphology did not present any relevant feature and the obtained images exhibit a flat surface and neither particles nor clusters were observable (images are not shown in this manuscript). This is because polymers are compatible and one phase is formed during the preparation process (the FTIR spectra demonstrated all the characteristic peaks of FUC, CHI, and PVA during the blending process and revealed that FUC was successfully coupled to CHI/PVA material [36]). On the other hand, cross-sectional images (Figure 3) showed that FUC creates a porous structure. This is caused by the strong ionic interaction of CHI positively charged amino groups with FUC negatively charged sulfate groups, which caused precipitation of the polymer solutions and formation of porous networks. The structure is considered as appropriate for adhesion and cell proliferation [66]. 

### 3.5. Degree of Swelling and Solubility

Materials in contact with living organisms require the ability to conduce and store water as an essential process to achieve proper cell signaling and nutrition [67]. For that reason, it is crucial to determine the degree of swelling and solubility of the obtained BAPM. The results are shown in Table 3. All the films swelled rapidly and reached equilibrium within the first 30 min of the experiment although relevant differences are caused by the presence of distinct components. PVA exhibits the highest degree of swelling because of the presence of hydrophilic groups, which are available for forming hydrogen bonding with water. Interactions between hydroxyl groups of PVA and amino or hydroxyl groups of CHI reduce the number of interactions between –OH groups and water [68], reducing the overall hydrophilicity of the system. Moreover, as the material was crosslinked and plasticized, there was a reduction of these groups and there were variations in the swelling. Although a reduction of swelling in systems made of PVA and CHI has been reported, and this is associated with the increase in the CHI ratio, in this study, PVA and CHI reacted with glutaraldehyde, and therefore the cross-linked blend becomes less capable of hydrogen bonding with water molecules due to acetalization and the formation of the Schiff base, which resulted in a decreased degree of swelling at equilibrium [69]. 

The presence of FUC increased the swelling of the material. The addition of negatively charged FUC increases the availability of free functional groups in the PVA/CHI/FUC blend. Hence, it exhibited a higher degree of swelling in comparison to the PVA/CHI material [36]. Although the swelling behavior of samples containing FUC is higher compared to the samples with its absence, the presence of plasticizer and crosslinker diminished the swelling as a result of the decline in the hydroxyl and amino groups. The surface generally increases upon swelling of the film, which makes it suitable for more cell adhesion and infiltration. Indeed, the presence of FUC in the BAPM resulted in increased surface area (rugosity on the surface), as was evidenced in SEM images. The water retention ability of the samples containing FUC was comparatively less than samples without FUC. This may be due to the fact that unbound water molecules are easily removed from the surface of FUC samples [70]. 

The water absorption capacity is increased with an increase in the ratio of CHI/FUC [71], however the presence of PVA is dominant as it has a high preference for water molecules due to the hydrophilic groups in the polymer. The addition of negatively charged FUC increases the availability of free functional groups in the blends, therefore, the swelling behavior of a material containing FUC is higher compared to a material without FUC. The presence of FUC in the films increases the surface area, which increases cell adhesion and infiltration [36]. 

### 3.6. Cell Proliferation (MTT Assay)

**Figure 4:**
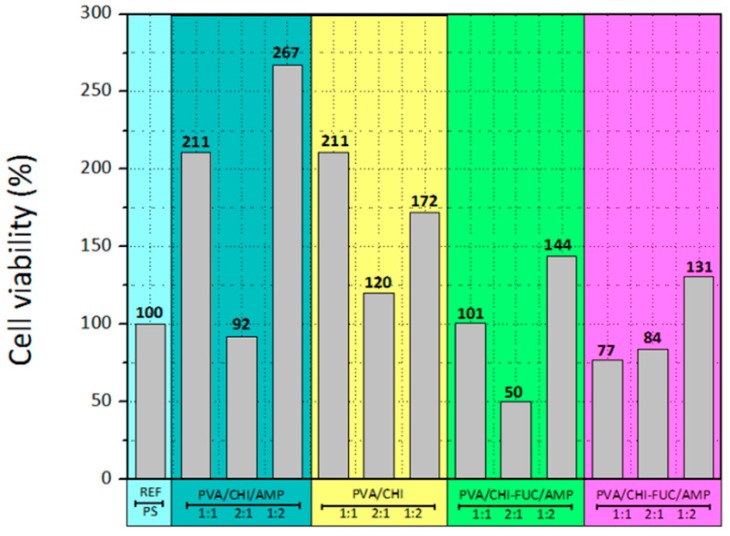
Cell viability of the BAPM (MTT assay).

Stability is a crucial factor for cell adhesion and proliferation. These cells behave in culture in a similar way as they do in vivo, migrating towards the air interface to form the epithelial surface. The cells’ behavior on the prepared films was evaluated by MTT assay and the results are given in Figure 4. After three days in culture, the results showed patterns that should be taken into account; for instance, systems 1:1 and 1:2 have higher proliferation than the samples where PVA is the main material of the formulation. In fact, the highest proliferation rates are on the 1:2 matrices. 

The antibiotic seems to have no adverse effect on cell growth, since the system PVA/CHI/AMP evinced higher proliferation than the sample without ampicillin. The histograms show that the most thriving sample in terms of cell viability was the PVA/CHI/AMP 1:2. On the other hand, FUC seems to have a detrimental effect, owing to the low proliferation rates when this polysaccharide was added. Indeed, the lowest proliferation rates are observed on the films 2:1 with FUC. It should be noted that cell proliferation is highly reliant on surface topography, surface crystallinity, hydrophilic/hydrophobic character, roughness and chemical compositions. As mentioned above, the systems with FUC have porous structures and solid particles across the films, which are counterproductive for cell growth. Fibroblast tends to grow efficiently on hydrophilic surfaces, and their attachment is mainly related to carbonyl and carboxyl groups along with hydrogen bonding and van der Waals forces, which reinforce the linking between cell and films. This information is in agreement with the spectroscopic results, since polar entities are the mainly organic functionalities of the studied systems [72,73]. 

## 4. Conclusions

Bioartificial polymeric materials are considered as a new class of constituents that combine the appropriate mechanical properties of artificial polymers with the satisfactory biological features of natural polymers. Thus, this research examined the development of a BAPM made of PVA, CHI, and FUC, and the incorporation of AMP as an antibacterial agent. The prepared films were tested and it was elucidated that the BAPM has potential for cell regeneration in vitro. The characterization techniques used here indicated that PVA brings water resistibility to the system, whereas CHI/FUC are responsible for creating a porous microstructure, which allows cells to adhere and grow within the matrix. The obtained information indicated that PVA, CHI, and FUC are compatible, as evidenced in FTIR spectra, as well as in SEM images. 

Although PVA/CHI films behaved better than PVA/CHI/FUC in terms of cell viability, FUC does not inhibit cell growth. On the other hand, a higher concentration of CHI/FUC displayed better properties for cell regeneration, which is an indication that the inherent attributes of these natural polymers favor interactions between living organism, and therefore, it is plausible to assume that the obtained film is a credible alternative for cell cultures. 

Although the presence of AMP reduces cell growth, the obtained results are promising because even though the obtained values are smaller, the potential to effectively control bacteria colonization brings alternative benefits to the film. Thus, these biomaterials are integral to the future of disciplines such as tissue engineering, and the ability to tailor cell regeneration is a desirable property in systems with potential use in living organisms. 

## Figures and Tables

**Table 1:** List of the prepared BAPMs and the ratio of all the components.

Description
PVA
CHI
PVA/CHI/AMP 1:1
PVA/CHI/AMP 2:1
PVA/CHI/AMP 1:2
PVA/CHI 1:1
PVA/CHI 2:1
PVA/CHI 1:2
PVA/CHI/FUCOIDAN/AMP 1:1
PVA/CHI/FUCOIDAN/AMP 2:1
PVA/CHI/FUCOIDAN/AMP 1:2
PVA/CHI/FUCOIDAN 1:1
PVA/CHI/FUCOIDAN 2:1
PVA/CHI/FUCOIDAN 1:2

Note: The ratio indicates the relationship between synthetic (PVA) and natural (both CHI/FUC) polymers.

**Table 2:** List of the XPS spectra given in atomic percent of all the components.

Samples	C1s%	O1s%	N1s%	S2p%
PVA/CHI/AMP 1:1	69.3	26.0	4.6	0.1
PVA/CHI/AMP 2:1	77.1	21.3	1.6	0.1
PVA/CHI/AMP 1:2	70.3	24.7	4.8	0.2
PVA/CHI 1:1	67.1	28.1	4.7	-
PVA/CHI 2:1	70.0	26.2	3.7	-
PVA/CHI 1:2	67.6	27.5	4.9	-
PVA/CHI/FUC/AMP 1:1	69.2	26.3	4.4	0.2
PVA/CHI/FUC/AMP 2:1	72.5	25.6	2.0	-
PVA/CHI/FUC/AMP 1:2	69.8	25.9	4.4	-
PVA/CHI/FUC 1:1	70.2	26.2	3.6	0.1
PVA/CHI/FUC 2:1	68.6	27.0	4.1	0.2
PVA/CHI/FUC 1:2	75.7	20.8	3.3	0.1

**Table 3:** Degree of swelling and solubility degree for the prepared BAPM.

MATERIAL	Degree of Swelling after 6 h [%] (SD)	Average Weight Loss [%] (SD)
PVA	1652 ± 337	36 ± 10
CHI	354 ± 45	32 ± 3
PVA/CHI/AMP 1:1	1364 ± 258	49 ± 7
PVA/CHI/AMP 2:1	1448 ± 148	52 ± 8
PVA/CHI/AMP 1:2	1301 ± 225	35 ± 10
PVA/CHI 1:1	1160 ± 244	52 ± 5
PVA/CHI 2:1	1263 ± 344	61 ± 3
PVA/CHI 1:2	1032 ± 108	36 ± 8
PVA/CHI/FUCOIDAN/AMP 1:1	1324 ± 208	36 ± 5
PVA/CHI/FUCOIDAN/AMP 2:1	1342 ± 76	54 ± 13
PVA/CHI/FUCOIDAN/AMP 1:2	1169 ± 303	31 ± 10
PVA/CHI/FUCOIDAN 1:1	1159 ± 173	36 ± 3
PVA/CHI/FUCOIDAN 2:1	1414 ± 404	38 ± 12
PVA/CHI/FUCOIDAN 1:2	1227 ± 44	38 ± 6
